# A chromosome-level genome sequence assembly of the red raspberry (*Rubus idaeus* L.)

**DOI:** 10.1371/journal.pone.0265096

**Published:** 2022-03-16

**Authors:** Jahn Davik, Dag Røen, Erik Lysøe, Matteo Buti, Simeon Rossman, Muath Alsheikh, Erez Lieberman Aiden, Olga Dudchenko, Daniel James Sargent

**Affiliations:** 1 Department of Molecular Plant Biology, Norwegian Institute of Bioeconomy Research, Ås, Norway; 2 Graminor Breeding Ltd., Ås, Norway; 3 Department of Agriculture, Food, Environment and Forestry (DAGRI), University of Florence, Florence, Italy; 4 Department of Plant Sciences, Norwegian University of Life Sciences, Ås, Norway; 5 The Center for Genome Architecture, Baylor College of Medicine, Houston, Texas, United States of America; 6 Center for Theoretical Biological Physics and Department of Computer Science, Rice University, Houston, Texas, United States of America; 7 UWA School of Agriculture and Environment, The University of Western Australia, Crawley, Australia; 8 Broad Institute of MIT and Harvard, Cambridge, Massachusetts, United States of America; 9 Shanghai Institute for Advanced Immunochemical Studies, Shanghai Tech, Pudong, China; 10 Department of Genetics, Genomics and Breeding, NIAB-EMR, East Malling, United Kingdom; 11 Natural Resources Institute, University of Greenwich, Chatham Maritime, United Kingdom; Virginia Polytechnic Institute and State University, UNITED STATES

## Abstract

*Rubus idaeus* L. (red raspberry), is a perennial woody plant species of the Rosaceae family that is widely cultivated in the temperate regions of world and is thus an economically important soft fruit species. It is prized for its flavour and aroma, as well as a high content of healthful compounds such as vitamins and antioxidants. Breeding programs exist globally for red raspberry, but variety development is a long and challenging process. Genomic and molecular tools for red raspberry are valuable resources for breeding. Here, a chromosome-length genome sequence assembly and related gene predictions for the red raspberry cultivar ‘Anitra’ are presented, comprising PacBio long read sequencing scaffolded using Hi-C sequence data. The assembled genome sequence totalled 291.7 Mbp, with 247.5 Mbp (84.8%) incorporated into seven sequencing scaffolds with an average length of 35.4 Mbp. A total of 39,448 protein-coding genes were predicted, 75% of which were functionally annotated. The seven chromosome scaffolds were anchored to a previously published genetic linkage map with a high degree of synteny and comparisons to genomes of closely related species within the Rosoideae revealed chromosome-scale rearrangements that have occurred over relatively short evolutionary periods. A chromosome-level genomic sequence of *R*. *idaeus* will be a valuable resource for the knowledge of its genome structure and function in red raspberry and will be a useful and important resource for researchers and plant breeders.

## Introduction

Red raspberries (*Rubus idaeus* L.) are edible berries composed of an amalgam of drupelets which are prized for their sweet, delicate taste and pleasant aroma. They are small, usually red berries that detach easily from their receptable when ripe and have a rich nutrient and bioactive composition, containing amongst other compounds polyphenolics, such as ellagitannins and anthocyanins [1]. The genus *Rubus* is a member of the morphologically diverse Rosaceae family, which comprises some 90 genera, and includes other economically important fruit and ornamental species including strawberry, blackberry, apple, pear, cherry, almond and peach. *Rubus* belongs to the Rosaceae sub-family Rosoideae [2], which is characterised by a base chromosome number of *x* = 7, and contains the other economically important genera *Fragaria* (the strawberries) and *Rosa* (the roses). The majority of Rosoideae species are shrubs or perennial herbs.

The European red raspberry, *R*. *idaeus*, is distributed throughout the temperate regions of the Northern hemisphere and is cultivated globally for its fruit, which is sold primarily as a fresh commodity, but which is also used extensively in processing. In total, 822,493 tonnes of raspberries were produced globally from a total growing area of 127,578 Ha in 2019 (http://www.fao.org/faostat/en/#data/QC), making it one of the most economically valuable soft fruits in commercial cultivation. Raspberries are often transported significant distances from production areas to consumer markets, with fruit produced in Mexico sold by retailers in the United States of America, and Spanish fruit exported to the UK and the Nordic countries.

Producers therefore require varieties with good shelf-life and shipping characteristics, whilst consumers demand higher quality berries with enhanced flavour and appearance. As such, there are a number of well-established public and private breeding programs worldwide with fruit quality, yield and storage properties, as well as pest and disease resistance, being primary breeding targets [3]. In recent years, significant progress has been made with the release of superior varieties from several programs that have increased the market share of red raspberry in the global soft fruit market.

Molecular genetics and genomics research into agronomic traits in red raspberry has been developing steadily and the genetic control of several traits, including annual fruiting [4] and fruit colour [5], are beginning to be elucidated in the species. Indeed, several of the breeding programs that exist globally are beginning to implement molecular tools for selection of key traits of agronomic importance. Early red raspberry molecular genetics research yielded a suite of tools that were used to develop genetic linkage maps of the species [6], including a sequence-based linkage map composed of SNP markers constructed using genotyping-by-sequencing [7]. However, despite these early advances, and a relatively small genome size of below 300 Mbp [8], the pace of development of genomics resources for red raspberry have fallen behind other closely related species. The genome of *Fragaria vesca* (the woodland strawberry) was the first plant genome to be sequenced and assembled entirely using short-read sequencing data [9] and more recently, a chromosome-length sequence was produced using long-read sequencing technology [10]. The genomes of other members of the Rosaceae family have also been sequenced, including most notably apple [11, 12], peach [13] and rose [14]. Furthermore, the genome sequence of several close relations of *R*. *idaeus*, *R*. *occidentalis* (black raspberry), and *R*. *chingii* have recently been published [15–17]. To date however, no genome sequence data for red raspberry is available in public data repositories.

Here, we present a chromosome-length genome sequence of the red raspberry (*R*. *idaeus*) variety ‘Anitra’ assembled using PacBio long read single-molecule real-time (SMRT) sequencing and scaffolded using Hi-C sequence data. The developed genome sequence is 291.7 Mbp in length, with 247.5 Mbp (84.8%) incorporated into seven sequencing scaffolds with an N50 of 34.5 Mbp. The seven scaffolds were anchored to the previously published genetic linkage map of [7] and were shown to correspond with a high degree of synteny to the seven linkage groups of that map. Gene prediction yielded 39,448 protein coding genes from the assembled sequence data. BUSCO was used to evaluate the completeness and integrity of the predicted gene set and 91.3% of BUSCO genes were functionally annotated.

## Materials and methods

### Plant material

The *R*. *idaeus* (red raspberry) cultivar ‘Anitra’, sourced from Graminor Breeding AS was used for genome sequencing and assembly. Leaf material was harvested from a single plant of this cultivar grown at the Njøs Fruit and Berry Centre, Leikanger, Norway.

### Genome size estimation

Flow cytometry was performed from young, unexpanded *R*. *idaeus* ‘Anitra’ leaves in biological triplicate using a *Vinca minor* internal standard (DNA content 1.52 pg/2C), as well as a standard with a known genome size of *Fragaria vesca* ‘Hawaii 4’ as a member of the Rosoideae sub-family.

### DNA extraction and genomic DNA sequencing

Young, unexpanded leaves of the *R*. *idaeus* cultivar ‘Anitra’ were harvested, flash frozen in liquid nitrogen and stored at −80°C prior to DNA extraction. DNA for whole-genome sequencing with the PacBio RSII sequencing platform was extracted from the leaf tissue samples using a genomic DNA miniprep protocol originally developed for tobacco leaf, modified from [18] in combination with Qiagen genomic 500 tips according to the manufacturer’s recommendation (Qiagen, Oslo, Norway). DNA for short-read sequencing on the Illumina HiSeq platform was extracted using the Qiagen Plant DNA mini kit (Qiagen, Oslo, Norway) according to the manufacturer’s recommendations and used for sequencing library prep.

A PacBio Sequel II library was constructed with a 20 kb insert size using a SMRTbell^™^ Template Prep Kit 1.0-SPv3 according to the manufacturer’s recommendations (Pacific Biosciences Inc., Menlo Park, CA, USA), and a final size selection step was performed using BluePippin (Sage Science Inc., Beverly, MA, USA) with a 9 kb cut-off. The library was sequenced by the Norwegian Sequencing Centre (Oslo, Norway) on the RSII platform using P6 C4 chemistry with a 360-minute movie time in 14 SMRT cells. A 250 bp paired-end insert library was constructed with an insert size of 350 bp using the TruSeq DNA PCR-Free Library Preparation Kit (Illumina, San Diego, CA, USA) according to the manufacturer’s recommendations and was sequenced using the Illumina HiSeq4000 sequencing platform by the Norwegian Sequencing Centre (Oslo, Norway).

Leaf tissue from the cultivar ‘Anitra’, prepared for Hi-C sequencing by Phase Genomics (Seattle, WA, USA), was fixed in 1% (vol/vol) formaldehyde and was prepared as previously described by [19]. The cross-linked DNA was subsequently digested overnight with the MboI restriction enzyme and the resultant sticky ends were biotinylated and proximity-ligated to form chimeric junctions which were then size-enriched and physically sheared to 350 bp. The chimeric fragments, which represented the original cross-linked long-distance physical interactions within the genome, were used to produce paired-end sequencing libraries using the TruSeq DNA PCR-Free Library Preparation Kit (Illumina, San Diego, CA, USA), and 150-bp paired-end reads were generated using the Illumina HiSeq4000 platform.

### Genome sequence assembly

The long-read genome sequence data generated using the PacBio Sequel platform, was used to perform a contig-scale assembly of the *R*. *idaeus* ‘Anitra’ genome using the Canu assembler [20] with default parameters. Two rounds of polishing using the Illumina short-read sequence data was performed using Pilon version 1.23 [21] with default parameters. Following assembly, heterozygous regions in the assembly were identified and removed using Purge Haplotigs v1.1.0 [22], with a 50% cut-off for identifying contigs as haplotigs.

A chromosome-level assembly of the ‘Anitra’ genome was then produced by the DNA Zoo Consortium using the Hi-C reads. Reads were quality checked to remove duplicates and aligned to the Purge Haplotigs Pac Bio assembly using Juicer v1.6.2 [23]. The 3D de novo assembly (3D-DNA) pipeline [24] was used to perform error-correction, anchoring, ordering and orienting of the draft assembly and 500 bp gapped Ns were inserted between contigs. The Hi-C assembly was then reviewed and polished using Juicebox Assembly Tools [25] and contact maps visualizing the alignments with respect to the Purge Haplotigs assembly were produced.

All raw sequencing data and the genome assembly of *R*. *idaeus* presented here are available at the NCBI and can be accessed with Bioproject ID XXXXXXX. Interactive Hi-C contact maps of the ‘Anitra’ genome sequence assembly are available via the www.dnazoo.org website (https://www.dnazoo.org/assemblies/Rubus_idaeus). The ‘Anitra’ genome sequence can also be accessed from the Genome Database for Rosaceae (https://www.rosaceae.org; [26]).

### Gene prediction and annotation

A repeat library was generated from the pseudo chromosomes using RepeatModeler [27] and repeat sequences and low complexity DNA sequences were identified and soft-masked using RepeatMasker [28] implemented in OmicsBox [29]. Gene prediction was then performed with Augustus v3.3.3 [30] using *F*. *vesca* v4.0.a2 proteins as a training set on the repeat-masked raspberry genome. The accuracy and completeness of the gene predictions was refined with Augustus v3.3.3 [30] using the *F*. *vesca* v4.0.a1 genome and the *F*. *vesca* v4.0.a2 proteins [31] following the methodology of [30]. Distribution of genes across the ‘Anitra’ genome was plotted and visualised using DensityMap [32].

Protein coding gene annotation completeness was determined using the BUSCO v4.1.4 pipeline [33] with default parameters using the gene families set defined for the embryophyta odb10 lineage. CD-HIT Protein v4.6.4 [34] was used with default parameters to estimate the proteome redundancy. Homology of the predicted protein-coding gene set was determined by pairwise sequence comparison using BLAST+ blastp-fast algorithm [35] against the NCBI nr, SwissProt, RefSeq, TrEMBL and Araport11 protein databases with an expectation value cutoff of 1e-6. BLAST+ analyses were launched through the Galaxy platform [36] (accessed 07.03.2021) using locally installed databases except Araport11, which was downloaded from The Arabidopsis Information Resource (TAIR, https://www.arabidopsis.org/). Functional annotation of the predicted proteins was performed using InterProScan v5 [37] to assign InterPro domains and Gene Ontology (GO) terms, and BlastKOALA v2.2 [38] and eggNOG-mapper v2 [39] for KEGG ortholog and KEGG pathway mapping, respectively.

### Synteny analysis

Synteny to the *R*. *idaeus* ‘Heritage’ genetic linkage map of [7] was determined by comparing the positions of the 4,521 molecular markers mapped to that linkage map with their physical positions on the ‘Anitra’ chromosome-length assembly using blastn [40]. Plots of the genetic vs. physical positions of each of the mapped genetic markers were produced using only the markers that were significantly associated with the expected ‘Anitra’ pseudochromosome in the assembly. Synteny to other publicly available Rosoideae genome sequences was determined using MUMmer4 [41] running default parameters and data were plotted using the R-script found at https://github.com/jmonlong/Hippocamplus/issues/2 with minor modifications. The genome sequences used for synteny comparisons were those of *R*. *occidentalis* v3.0 [15], *R*. *chingii* v1.0 [17], *Fragaria vesca* v4.0 [10] and *Rosa chinensis* ‘Old Blush’ v2.0 [14], all of which were downloaded from the data repository on the Genome Database for Rosaceae (https://www.rosaceae.org; [26]).

## Results and discussion

### Chromosome-length assembly of the red raspberry genome

In this study, a chromosome-level *R*. *idaeus* genome based on PacBio and Hi-C sequencing of DNA from the cultivar ‘Anitra’ was produced (Table 1). Most (84.3%) of the assembled sequence data were contained in seven Hi-C sequencing scaffolds (psudochomosomes) that represented the seven base chromosomes of the *R*. *idaeus* genome.

**Table 1:** Summary statistics for the genome sequence of *R*. *idaeus* cv. ‘Anitra’.

*R*. *idaeus* ‘Anitra’ genome sequence	Pac Bio assembly	Hi-C assembly–all (including embedded gaps)	Hi-C assembly–seven pseudo-chromosomes (including embedded gaps)
Estimated genome size (flow cytometry)(Mbp)	293.4	293.4	293.4
Assembled genome size (bp)	291,080,678	291,685,178	247,480,545
Assembly rate	99.2%	99.2%	84.3%
Number of contigs	2,350	2,370	1,175
Largest contig (bp)	4,726,662	-	-
Number of scaffolds	2,350	1,161	7
Largest scaffold (bp)	-	42,972,798	42,972,798
Contig N50 (bp)	241,822	-	-
Contig NG50 (bp)	340,977	-	-
Scaffold N50	-	34,491,998	34,491,998
Scaffold NG50 (bp)	-	35,735,390	35,735,390

Flow cytometry returned a DNA content of 0.60 pg/2C for the ‘Anitra’ genome. The genome of *F*. *vesca* ‘Hawaii 4’ was included as a control and returned an average DNA content of 0.51 pg/2C, which was in agreement with a previously published flow cytometry size estimation for *F*. *vesca* ‘Hawaii 4’ [42]. Previous studies of the genome size of *R*. *idaeus* using flow cytometry reported the DNA content to be 0.58 pg/2C [8] and as such, the results presented here demonstrate that the genome of ‘Anitra’ is diploid and contains approximately the same DNA content of previously-studied accessions of *R*. *idaeus*. Using the calculation of [43], 1 pg of genomic DNA is equivalent to 978 Mbp of DNA sequence, and thus the genome size of *R*. *idaeus* ‘Anitra’ was calculated to be 293.4 Mbp, indicating 99.2% of the genome sequence had been incorporated in the initial assembly (Table 1).

Sequencing of Illumina and PacBio sequencing libraries yielded a total of 87.2 Gb of genome sequence data, comprising 66.3 Gb of Illumina sequence data (245×) and 20.9 Gb PacBio data (77×). The long PacBio subreads in the PacBio dataset had a mean N50 length of 13.6 kb. A total of 2,182,681 PacBio sub-reads were produced with an average sub-read length of 9,594 bp and an N50 sub-read length of 13,569 bp. The total number of sub-read bases sequenced was 20.9 Gb, with a total of 14. 5 Gb of unique bases sequenced. Furthermore, 87.2 Gb of Hi-C data was produced for long-range scaffolding of the genome assembly.

**Fig 1:**
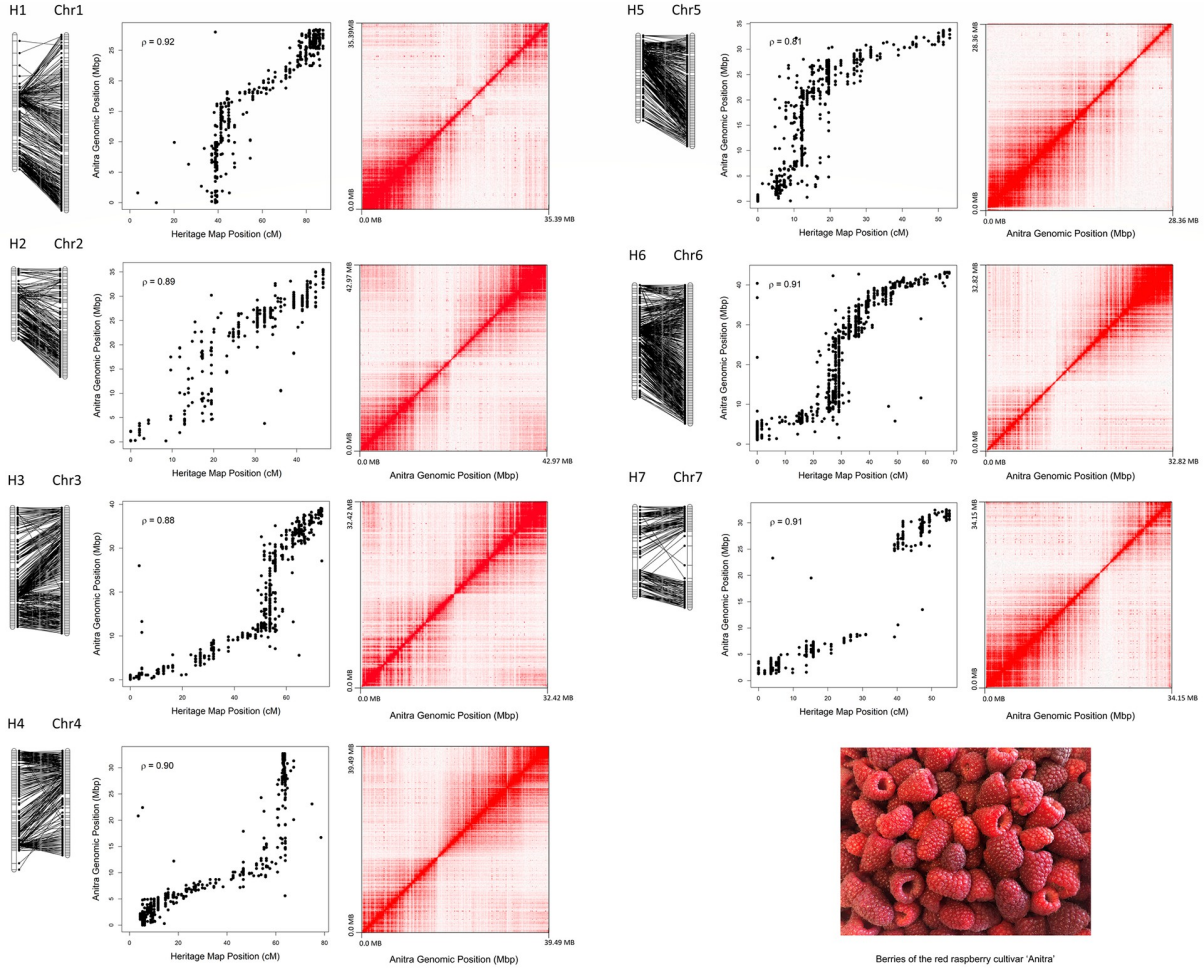
The seven chromosomes of the genome of *R*. *idaeus* ‘Anitra’. Genetic and physical positions of the 4,948 molecular markers on the reconstructed chromosomes of the ‘Anitra’ genome and the ‘Heritage’ genetic linkage map of [7] (left panels). Scatter plots showing the physical positions of the same molecular markers on each ‘Anitra’ chromosome (Chr) (*x* axis) in relation to the ‘Heritage’ map positions (*y* axis) where Rho (ρ) is the Pearson correlation coefficient for the associations (central panels). Hi-C intrachromosomal contact maps for each ‘Anitra’ chromosome are given in the right panels. The intensity of pixels represents the proportion of Hi-C links within 400-kb windows along each chromosome plotted on a logarithmic scale.

The initial assembly of the PacBio data returned a genome sequence with a total length of 291,080,678 bp, contained in 2,350 contigs with an N50 of 241,822 bp, and a longest contig length of 4,726,662 bp. Scaffolding of the assembly using the Hi-C data yielded a high-quality chromosome-level genome assembly of the *R*. *idaeus* cultivar ‘Anitra’. Ten contigs were identified as erroneously assembled and were each split into two meaning 2,370 contigs were available for scaffolding. The scaffolded Hi-C assembly contained a total of 291,685,178 bp of sequence including embedded 500 bp gaps between contigs in 1,161 scaffolds with an N50 of 34,491,998 bp and an NG50 of 35,735,390 bp (Table 1). The largest seven scaffolds, incorporated 1,175 of the original Pac Bio contigs and represent the seven expected chromosomes of the *R*. *idaeus* genome. In total they contained 247,480,545 bp of sequence data including embedded gaps, with the largest scaffold containing 42,972,798 bp and the shortest containing 28,368,965 bp. The seven pseudochromosomes were numbered according to the nomenclature of the other sequenced Rosoideae genomes that began with the *F*. *vesca* ‘Hawaii 4’ genome [9] (Fig 1).

Initially, genomes sequenced with long-read sequencing chemistry significantly improved assemblies based on short-read sequence data alone, i.e. the genome of a close relative of *R*. *idaeus*, *Potentilla micrantha*, where the addition of long PacBio subreads significantly enhanced contig lengths and eliminate many gaps within scaffolds, but the resultant genomes still remained highly fragmented [42]. Other *R*. *idaeus* genome sequences have previously been reported in the literature, including a fragmented assembly of the *R*. *idaeus* cultivar ‘Glen Moy’ [44], and the draft assembly of the cultivar ‘Joan J’, which consisted of 300 Mbp of sequence in 2,145 sequencing scaffolds and a genome completeness of 95.3% based on BUSCO [45]. Of the 300 Mbp of sequenced genome, 240 Mbp (80%) was anchored to seven pseudochomosomes via the the genetic linkage maps of [7], lower than the 85% of sequence data scaffolded using Hi-C in this investigation. However, the sequences of the other reported *R*. *idaeus* genomes are not currently available in public repositories.

The recent development and implementation of Hi-C sequencing has significantly improved genome assemblies and has permitted chromosome-length assemblies of the genomes of many minority species, including several relatives of *R*. *idaeus*, most notably the genome of *F*. *vesca* [10]. The implementation of Hi-C in the assembly of the genome reported here for the red raspberry cultivar ‘Anitra’ permitted a chromosome-length assembly of the genome that represents a significant improvement over previously published *R*. *idaeus* genome assemblies. The initial release of the genome of a highly homozygous accession of *R*. *occidentalis* (black raspberry; [15]), a closely related species to *R*. *idaeus* had a total sequence length of 239.8 Mbp and a scaffold N50 of 353,335 bp. Scaffolds that were genetically anchored to seven pseudochromosomes [15]. Subsequent iterations of the genome improved the coverage and scaffold size and, as in this study, employed Hi-C to scaffold the genome into seven pseudochromosomes containing 223.8 Mb of sequence scaffolds (93.3% of the total sequence length). More recently, the genome of *R*. *chingii* [17] was sequenced using the Oxford Nanopore sequencing platform and Hi-C. The chromosome-length genome sequence spanned 231.21 Mb (scaffold N50 = 8.2 Mbp), of which 220.05 Mb were anchored to seven chromosomes using Hi-C.

The ‘Anitra’ sequence presented here is comparable in length to the genome assembly of ‘Joan J.’ based on previously-reported sequencing statistics for that genome [45], and the total ‘Anitra’ genome sequence length of 291.7 Mbp was similar to the genome size estimate based on flow cytometry (293.4 Mbp). A total of 247.5 Mbp (84.3%) of the genome were incorporated into seven pseudomolecules that represent the seven chromosomes, comparable to the sequence coverage of the black raspberry pseudochromosomes reported by [16], and as such the assembly presented here is a high quality chromosome-length draft genome sequence resource for the red raspberry research community.

### Gene prediction and annotation

**Fig 2:**
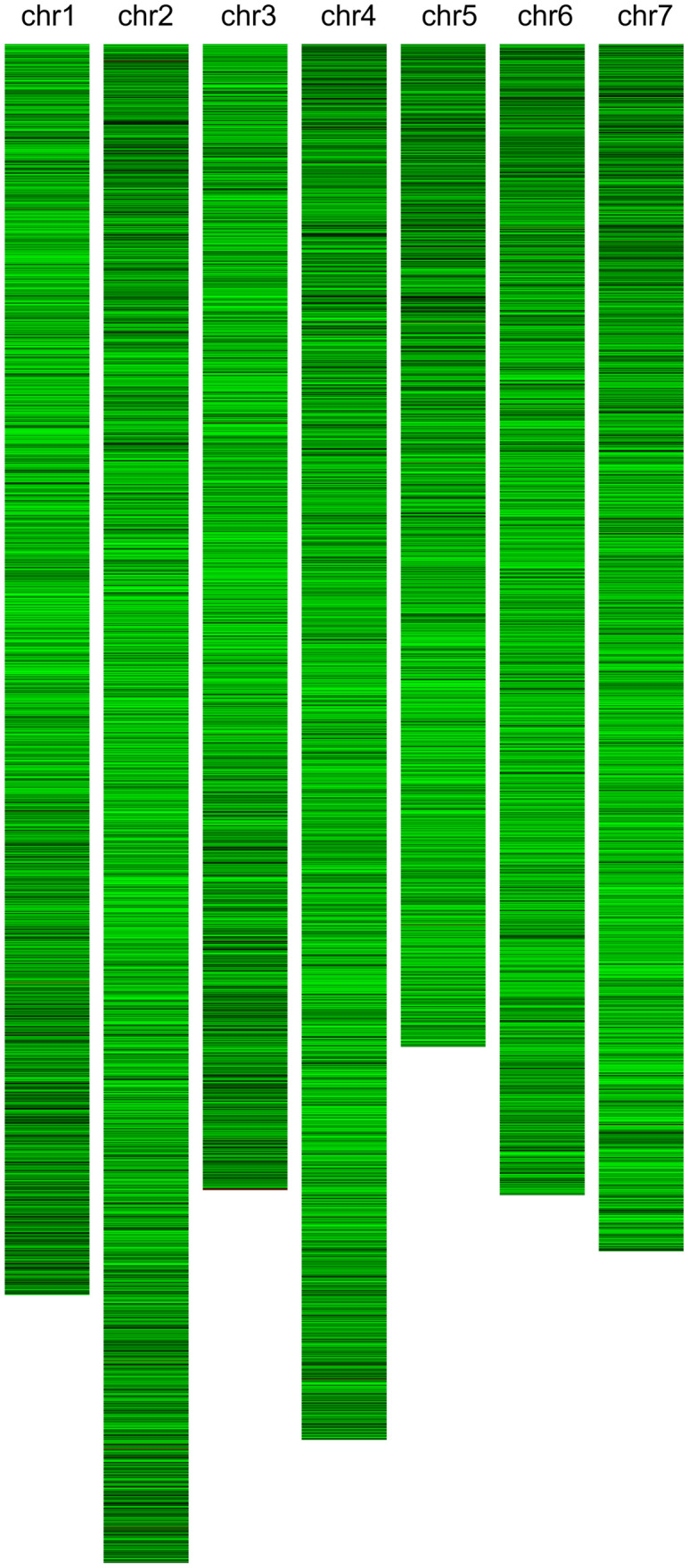
Visualisation of gene distribution and densities across the seven chromosomes of the *R*. *idaeus* ‘Anitra’ genome. The seven chromosomes are represented by the vertical strands, gene density is represented by the horizontal bands with a window of 10,000 bp. The darker the colour, the greater the density of genes.

A total of 39,448 protein-coding genes were identified in the *R*. *idaeus* ‘Anitra’ genome sequence, with a mean number of 4.52 exons and 3.52 introns per gene, and mean exon and intron lengths of 263 bp and 414 bp, respectively. Distribution of genes across the seven ‘Anitra’ chromosomes is shown in Fig 2. The completeness of the predicted proteome was evaluated with BUSCO. In total, 98.0% of the 1,614 groups queried were identified within the predicted proteome; 1,518 were complete and single copy, whilst 63 were complete and duplicated BUSCOs, 12 were fragmented and 21 were missing from the assembly. The analysis completeness score for the ‘Anitra’ genome was similar to those of other previously published genomes assembled using similar strategies [14, 45, 46], indicating that the ‘Anitra’ assembly was of good quality. Table 2 summarises the ‘Anitra’ genome sequence statistics alongside other sequenced genomes of the Rosoideae for which data are publicly available.

**Table 2:** Comparison of assembly statistics for *R*. *idaeus* cv. ‘Anitra’ and three closely-related Rosoideae genome assemblies.

	‘Anitra’	*R*. *chingii* [17]	*R*. *occidentalis* [15]	*F*. *vesca* [10]
Estimated genome size (flow cytometry)	293.4 Mb	-	293 Mb	254.28 Mb [42]
Assembled genome size	291.1 Mb	231.21 Mb	290.8 Mb	220.8 Mb
Number of contigs	2,370	-	235	61
Number of scaffolds	1,161	155	7	31
Number of contigs in pseudomolecules	1,175	-	235	-
contig N50	242.2 Kb	-	5.1 Mb	7.9 Mb
Scaffold N50	34.5 Mb	8.2 Mb	41.1 Mb	36.1 Mb
Assembly rate of genome (%)	99.2%	-	99.3%	99.8%
Number of chromosomes	7	7	7	7
Size of sequence anchored on chromosomes	247.5 Mb	220.05 Mb	290.8 Mb	220.8 Mb
Anchoring rate on chromosomes (%)	85%	95.17%	100%	100%
Average chromosome length	35.4 Mb	31.4 Mb	41.5 Mb	
Number of predicted protein-coding genes	39,448	33,130	34,545	28,588
Average coding sequence length	1,189 bp	2,803 bp	3,220 bp	1,475 bp
BUSCO score	98.0%	97.1%	94%	95%

Proteome redundancy revealed using CD-HIT grouped the 39,448 predicted raspberry proteins into 34,120 clusters based on sequence homology, indicating low proteome redundancy. The number of predicted proteins was comparable with those of the genomes of black raspberry (*R*. *occidentalis*) sequenced using similar methods [15], and *R*. *chingii* [17], as was the proteome completeness, indicating that the majority of coding part of the genome is represented in the assembly reported here. Within the 39,448 red raspberry protein-coding gene predictions, a total of 29,565 (75%), 25,168 (63.8%), 29,484 (74.7%), 22,194 (56.2%), and 28,358 (71.9%) returned ≥1 hit after the blastp analysis with nr, Araport11, RefSeq, SwissProt and TrEMBL databases as subjects, respectively. Functional annotation analyses assigned Interpro, GO, KEGG orthology and KEGG pathway terms to 25,342 (64.2%), 19,089 (48.4%), 10,474 (26.6%) and 7,984 (20.2%) protein-coding gene predictions, respectively. The best hits resulting from homology analyses against NCBI nr, SwissProt, RefSeq, TrEMBL and Araport11 protein databases are detailed in S1 Table, while the functional annotation results including InterPro domains assignment, Gene Ontology terms, KEGG orthologs and KEGG pathway mapping are detailed in S2 Table.

### Comparative analysis and synteny with other Rosaceous genomes

In total, 4,948 markers from the *R*. *idaeus* ‘Heritage’ linkage map of [7] returned significant associations with the seven ‘Anitra’ pseudochromosomes (Fig 1, S3 Table). A high degree of synteny was observed between the linkage groups of [7] and the seven ‘Anitra’ pseudochromosomes, with no major chromosomal rearrangements identified between the two datasets, indicating that the Hi-C pseudochromosomes has been assembled faithfully, and in accordance with genetic information previously published from the red raspberry genome. A Hi-C contact map for the whole genome is given in S1 File. Of the 1,195 contigs that were not incorporated into the seven pseudochromosomes of the ‘Anitra’ genome, 353, representing 20.7 Mbp of sequence were associated with molecular markers mapped to the ‘Heritage’ genome sequence. However, the majority of these contigs could not be unambiguously assigned to a single genetic region on the ‘Heritage’ linkage map, and as such, they are included in the v1.0 assembly release as unassigned contigs.

**Fig 3:**
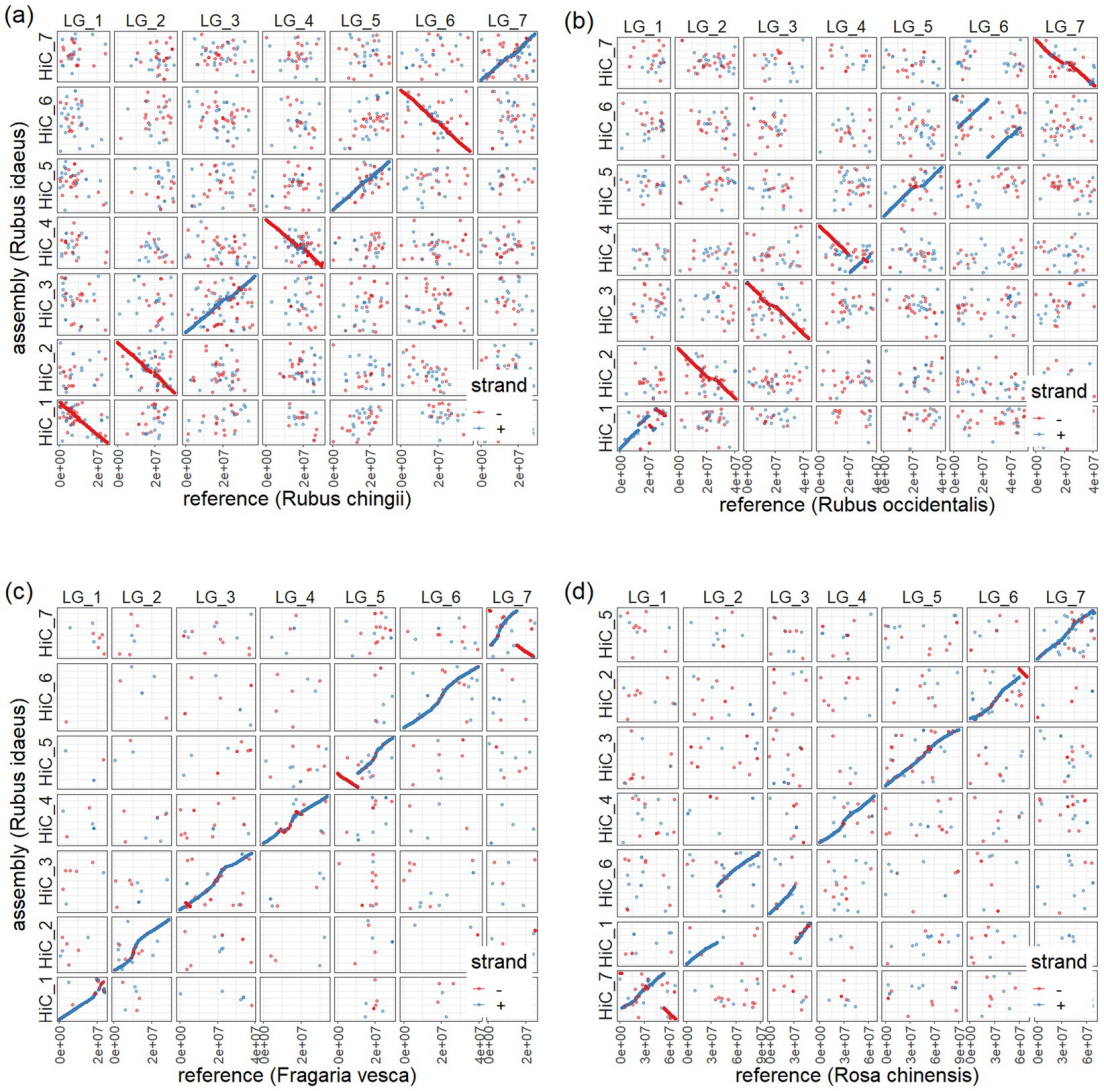
MUMMer plots of the macro-synteny between the *Rubus idaeus* ‘Anitra’ genome and (a) *R*. *chingii*; (b) *R*. *occidentalis*; (c) *Fragaria vesca* ‘Hawaii 4’; and (d) *Rosa chinensis* ‘Old Blush’.

The monophyly of the Rosoideae within the Rosaceae family was established by [47], with a subsequent reclassification by [48] establishing a single supertribe, the Rosodeae that contains all the Rosoideae genera for which genome sequences have been published, *Fragaria*, *Potentilla*, *Rosa* and *Rubus*. *Rubus* was shown by [48] to be one of the most basal genera within the Rosodeae supertribe and as a result, comparisons of the ‘Anitra’ genome to other sequenced Rosoideae genomes may identify significant chromosome-scale evolutionary events that have occurred during the evolution of genera and species within this group. A total of 30,036 loci were used for the comparison between the seven ‘Anitra’ pseudochromosomes and those of *R*. *chingii* [17], 25,845 with *R*. *occidentalis* [16], 12,516 with *Rosa chinensis* [14] and 8,361 with *F*. *vesca* ‘Hawaii 4’ v4.0 [10]. A high degree of synteny was observed between the ‘Anitra’ genome and the four previously published genomes (Fig 3), with all ‘Anitra’ chromosomes corresponding to a single orthologous chromosome in all the other species save for *R*. *chinensis*, where evidence for a chromosomal fusion-fission event was evident between the genomes of the two species. However, several large-scale chromosomal rearrangements were observed between the ‘Anitra’ genome and those of the other species. The rearrangements observed were not evident in the comparison of the ‘Anitra’ genome to the *R*. *idaeus* genetic linkage map of [7], indicating that these were not assembly errors, and that they actually represented real events in the evolutionary history of the species compared.

Almost complete macro-synteny was observed between the ‘Anitra’ and *R*. *chingii* genome sequences (Fig 3a) with all seven chromosomes corresponding to a single, complete chromosome between species and no large-scale rearrangements observed along the length of any of the seven chromosomes. Synteny was lower between the genomes of ‘Anitra’ and *R*. *occidentalis* (Fig 3b), with several small rearrangements observed on chromosome 1 between the two species, a large inversion on chromosome 4, and two large inversions originating from the same chromosomal breakpoint identified on chromosome 6, along with a smaller inversion towards the distal end of that chromosome. Several smaller inversions were also observed on chromosomes 4 and 7. These rearrangements were not evident in comparisons of the ‘Anitra’ genome with the *R*. *idaeus* linkage map of [7] or the genome of *R*. *chingii*, suggesting that they occurred in the *R*. *occidentalis* genome since the relatively recent split of *R*. *idaeus* and *R*. *occidentalis* from their common ancestor [49]. However, given the relatively small number of rearrangements observed over a much greater evolutionary time-frame between the ‘Anitra’ genome and the *Fragaria* and *Rosa* genomes, it would suggest that many of the large inversions observed may likely be errors in the *R*. *occidentalis* genome sequence assembly.

The ‘Anitra’ and *F*. *vesca* (Fig 3c) genomes displayed a high degree of synteny between pseudochromosomes, with just two large inversions observed on chromosomes 5 and 7, and smaller inversions observed on chromosomes 3 and 4. Evidence for some of these rearrangements was reported in previous studies of genetically mapped markers [50], in particular, the inversion on chr5. As with the comparison of the ‘Anitra’ genome to that of *R*. *occidentalis*, lower synteny was observed at the distal end of chromosome 1, however the major inversion events revealed in *R*. occidentalis were not observed in *F*. *vesca*, supporting the hypothesis that the observations of the *R*. *occidentalis* genome were a result of mis-assembly in that sequence. Overall, a higher frequency of macro-scale chromosomal rearrangements was observed between *R*. *occidentalis* since its split from a common ancestor with *R*. *idaeus* than between the *F*. *vesca* and *R*. *idaeus* genomes compared.

Between the ‘Anitra’ and the *R*. *chinensis* genomes (Fig 3d), evidence of two significant translocations was detected between chromosome 1 and chromosome 6, whilst a small distal inversion on chromosome 2 and a large inversion on chromosome 7 was observed. A previous study by [51] comparing 70 genetically-mapped markers with their physical positions on the *F*. *vesca* genome identified the same translocations between chromosome 1 and chromosome 6 revealed in the comparative analysis of the genome sequences of ‘Anitra’ and *R*. *chinensis* performed here.

### Conclusions

In this investigation, we have produced a high-quality chromosome-level genome sequence for the European red raspberry species *R*. *idaeus* cv. ‘Anitra’ along with a set of gene predictions. Comparison to previously published linkage maps [7] and the genome of a closely related species [17] demonstrated that the sequence presented is of high quality and represents the majority of the *R*. *idaeus* genome. The ‘Anitra’ genome presented here, and its associated gene predictions, will be a valuable tool for future genomics studies in red raspberry and will allow more detailed functional studies of genes of agronomic importance in the species. The genome sequence will help accelerate marker development and basic science in raspberry, assist in the identification of genes controlling economically important agronomic traits, and hasten efforts to deploy molecular tools. Ultimately, it will assist the breeding and selection of new red raspberry varieties optimised for the changing cultivation conditions facing the soft fruit industry.

## Supporting information

S1 Table Blastp analyses of NCBI nr, SwissProt, RefSeq, TrEMBL and Araport11 protein databases using the ‘Anitra’ gene predictions as queries. (XLSX)

S2 Table Functional annotation of the ‘Anitra’ gene predictions including InterPro domains, Gene Ontology terms, KEGG orthologs and KEGG pathway mapping analyses. (XLSX)

S3 Table Genetic and physical positions of the 4,948 molecular markers on the reconstructed chromosomes of the ‘Anitra’ genome and the ‘Heritage’ genetic linkage map of [7] along with their percentage identity returned following Blastn analysis. (XLSX)

S1 File A Hi-C contact map showing associations across the whole ‘Anitra’ genome sequence. (TIF)
