# Supplementary figures and images for: A chromosome-level genome sequence assembly of the red raspberry (Rubus idaeus L.)
Source: PLoS One. 2022 Mar 16;17(3):e0265096. doi: 10.1371/journal.pone.0265096 (PMC8926247; doi:10.1371/journal.pone.0265096)

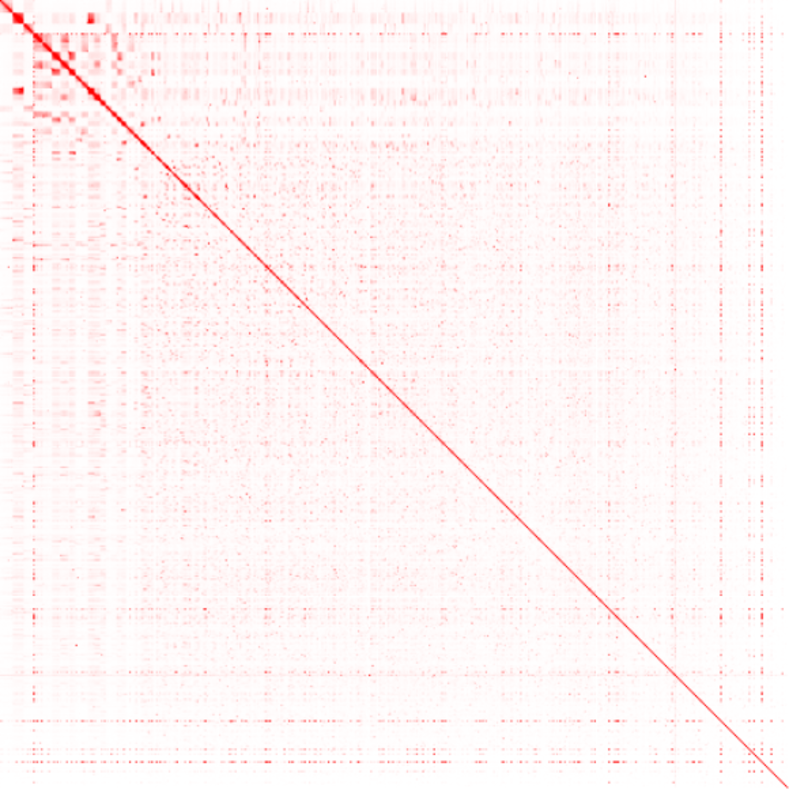

Supplement: S1 File — (TIF) [file pone.0265096.s004.tif]
